# Deoxynivalenol and Oxidative Stress Indicators in Winter Wheat Inoculated with *Fusarium graminearum*

**DOI:** 10.3390/toxins6020575

**Published:** 2014-02-07

**Authors:** Agnieszka Waśkiewicz, Iwona Morkunas, Waldemar Bednarski, Van Chung Mai, Magda Formela, Monika Beszterda, Halina Wiśniewska, Piotr Goliński

**Affiliations:** 1 Department of Chemistry, Poznań University of Life Sciences, Wojska Polskiego 75, Poznań 60-625, Poland; E-Mails: agat@up.poznan.pl (A.W.); monika.beszterda@up.poznan.pl (M.B.); 2 Department of Plant Physiology, Poznań University of Life Sciences, Wołyńska 35, Poznań 60-637, Poland; E-Mails: morkunas@jay.up.poznan.pl (I.M.); chungmai@up.poznan.pl (V.C.M.); formelamagda@o2.pl (M.F.); 3 Institute of Molecular Physics, Polish Academy of Sciences, Smoluchowskiego 17, Poznań 60-179, Poland; E-Mail: waldemar.bednarski@ifmpan.poznan.pl; 4 Department of Plant Physiology, Vinh University, Le Duan 182, Vinh City, Vietnam; E-Mail: chungmv@vinhuni.edu.vn; 5 Department of Genomics, Institute of Plant Genetics, Polish Academy of Sciences, Strzeszyńska 34, Poznań 60-479, Poland; E-Mail: hwis@igr.poznan.pl

**Keywords:** deoxynivalenol, semiquinone radicals, *Fusarium graminearum*, oxidative stress, winter wheat, zearalenone, ergosterol

## Abstract

This study comprises analyses of contents of mycotoxins, such as deoxynivalenol and zearalenone, as well as the level of oxidative stress in ears of a susceptible wheat cultivar Hanseat and cv. Arina, resistant to a pathogenic fungus *Fusarium graminearum*. Starting from 48 h after inoculation, a marked increase was observed in the contents of these mycotoxins in ears of wheat; however, the greatest accumulation was recorded in the late period after inoculation, *i.e.*, during development of disease. Up to 120 h after inoculation, in ears of both wheat cultivars, the level of deoxynivalenol was higher than that of zearalenone. The susceptible cultivar was characterized by a much greater accumulation of deoxynivalenol than the resistant cultivar. At the same time, in this cultivar, in the time from 0 to 72 h after inoculation, a marked post-infection increase was observed in the generation of the superoxide radical (O_2_^•−^). Additionally, its level, at all the time points after inoculation, was higher than in the control. In wheat cv. Arina, a markedly higher level of O_2_^•−^ generation in relation to the control was found up to two hours after inoculation and, next, at a later time after inoculation*.* In turn, the level of semiquinone radicals detected by electron paramagnetic resonance (EPR) increased at later culture times, both in cv. Hanseat and Arina; however, in infested ears of wheat, it was generally lower than in the control. Analysis of disease symptoms revealed the presence of more extensive lesions in ears of a susceptible wheat cv. Hanseat than resistant cv. Arina. Additionally, ergosterol level as a fungal growth indicator was higher in ears of susceptible wheat than in the resistant cultivar.

## 1. Introduction

Interactions of plants and their pathogenic fungi now constitute an interesting and rapidly developing field in plant science, with a significant impact on new strategies for plant protection. The plant response to infection is determined by the genetic background of the host, as well as the pathogen [1]. The type of induced response that is effective against a given pathogen varies, depending on the lifestyle of the pathogen [2]. Pathogens have devised different strategies to invade a plant, as well as to feed on and reproduce in the plant. Biotrophic pathogens need living tissue for growth and reproduction; in many interactions the tissue will die in the late stages of the infection (hemi-biotrophic pathogens). By contrast, necrotrophic pathogens kill the host tissue at the beginning of the infection and feed on the dead tissue [3].

As plants are confined to the place where they grow, they have to develop a broad range of defense responses to cope with pathogenic infections. Oxidative burst, a rapid, transient production of huge amounts of reactive oxygen species (ROS), is one of the earliest observable manifestations of a plant’s defense strategy [4,5,6]. Various aspects, mechanisms, and functions of the oxidative burst with generation of superoxide anions (O_2_^•−^) in plant cells, which is stimulated by active defense-inducing fungal infection or elicitor treatment, were reviewed mainly on the basis of experimental evidence obtained in different pathosystems [7,8,9]. Free radicals, including ROS, may function in defense through their direct toxicity to pathogens, or may activate various metabolic pathways. Enhanced generation of free radicals, such as ROS, plays a significant role especially at the early plant-pathogen interaction, whereas, at a later stage of the disease development—when not coordinated with an effective system of their removal—it may enhance destructive changes in plants and facilitate the spread of a pathogen [6,10,11]. Recently, concluding evidence suggests that the ROS network is essential to induce disease resistance [12]. On the other hand, investigations show also that necrotrophic pathogens can use oxidative processes during their attack and invasion of plant tissues [13]. Therefore, host cell death can occur through the action of fungal toxins and an oxidative burst generated by both the pathogen and the host [14].

This study, next to oxidative stress indexes indicating early defense responses of plants, also investigated the accumulation of mycotoxins formed by a pathogenic fungus *Fusarium graminearum*. Reverberi and co-workers reported that several secondary metabolites are synthesized by fungi during morphological and metabolic transitions when the accumulation of ROS occurs [15]. Plant compounds involved in plant-fungi interactions are able to interfere with mycotoxin biosynthesis in host tissues [16]. Mycotoxins are harmful and often carcinogenic secondary metabolites produced by a range of widespread fungi, including *Fusarium*. In general, they are low-molecular-weight compounds synthesized by filamentous fungi and are capable of causing disease and death in plants, animals and humans [17]. While in the literature there are many reports indicating high toxicity of mycotoxins, little is known about their role in plant-pathogen interactions. The relationship between the decrease in cell proliferation, the presence of oxidative stress generated by the enhancement of intracellular ROS production, and ROS-induced lipid peroxidation by mycotoxins is a priority direction of research [18]. *Fusarium* mycotoxins, currently considered of importance from the toxicological point of view, include zearalenone, trichothecenes and fumonisins, and their occurrence is now regulated by legal limits in all developed countries [19,20]. Among trichothecenes, deoxynivalenol (DON) is the most popular mycotoxin formed mainly by *Fusarium graminearum* and *F. culmorum* [19]. *Fusarium graminearum* is most common in moist and warm continental climates, such as Central and South-Eastern Europe, whereas *F. culmorum* is found more often in maritime and cooler European countries [21,22,23]. The primary sources of DON are cereals, including wheat, barley, maize, and oat [24,25]. Toxicity is associated with the presence of both 9, 10 double bond, 12, 13 epoxide group and varied substituent groups in the deoxynivalenol structure [26]. DON is responsible for the inhibition of protein biosynthesis, reduction of enzymatic activity, disturbance in cytoplasmic membrane permeability, and cell division disorders [26]. Another important mycotoxin, similar to DON, produced mainly by the same fungi, is zearalenone (ZON) [27].

The aim of the present study was to examine the interdependence between the level of oxidative stress and mycotoxin contents in ears of two winter wheat cultivars, *i.e.*, the susceptible cv. Hanseat and cv. Arina, resistant to a pathogenic fungus *Fusarium graminearum*. Therefore, the level of superoxide anion radical generation and concentrations of free radicals, such as semiquinones, were estimated in non-inoculated (control) and *F. graminearum* *-* inoculated ears of winter wheat. The semiquinone radicals analyzed in this study using electron paramagnetic resonance (EPR) spectrometry are among the relatively stable radicals that readily donate electrons to molecular oxygen (O_2_), forming O_2_^•^^−^. Moreover, changes in mycotoxin contents, such as deoxynivalenol and zearalenone, were determined in ears of the above-mentioned wheat cultivars. Additionally, disease symptoms were analyzed and ergosterol level as a fungal growth indicator was estimated in both wheat cultivars.

## 2. Results

### 2.1. Mycotoxin Contents

**Figure 1:**
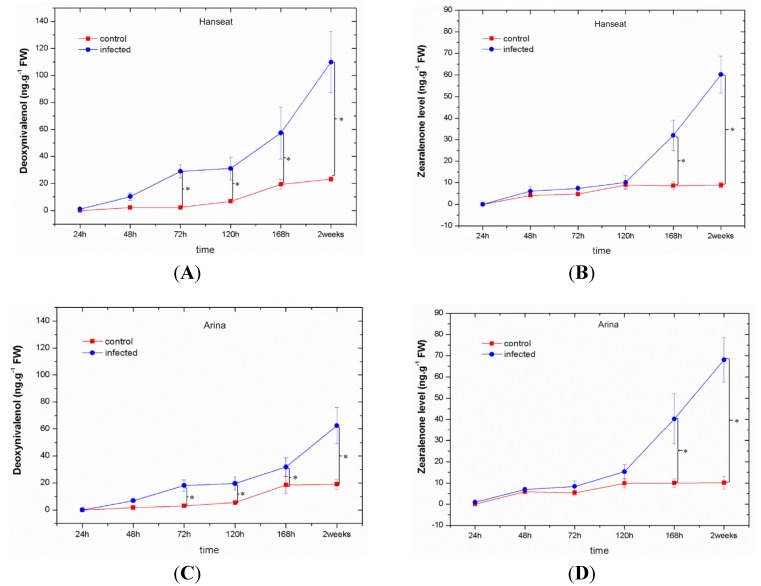
The effect of pathogenic fungus *Fusarium graminearum* on the content of mycotoxins, such as deoxynivalenol and zearalenone, in ears of a susceptible wheat cv. Hanseat (**A**,**B**) and resistant cv. Arina (**C**,**D**). Significant differences (*p* < 0.05) were observed between control and infected ears.

Note: * significant differences on figures using asterisks are shown.

Starting from 48 h after inoculation with a pathogenic fungus *F. graminearum* a marked increase was observed in the contents of mycotoxins, such as deoxynivalenol and zearalenone, in ears of wheat—both the susceptible cv. Hanseat and the resistant Arina (Figure 1). The highest accumulation of mycotoxins was recorded at late time points after inoculation (at 168 h and in week two), *i.e.*, during development of disease. Up to 120 h after inoculation in ears of both wheat cultivars, a higher level of deoxynivalenol (DON) was found in comparison to zearalenone (ZON). Analysis of variance (ANOVA) results showed that the differences in concentrations of DON and ZON in inoculated ears of wheat cultivars were highly statistically significant. It needs to be stressed that the susceptible cultivar (Figure 1A,B) was characterized by a much greater accumulation of deoxynivalenol than the resistant cultivar (Figure 1C,D). In the susceptible cultivar, the level of deoxynivalenol ranged from 1.1 to 109.88 ng g^−1^ FW, while in the resistant cultivar it was from 1.76 to 62.41 ng g^−1^ FW. Only at 168 h and in week two after inoculation in ears of the resistant wheat cv. Arina, zearalenone level was higher than that of deoxynivalenol (Figure 1C,D). ANOVA results showed that the differences in DON concentration in infected tissue of cultivars Hanseat/Arina and the control plants at 72, 120, 168 h, and two weeks were highly statistically significant (e.g., *p* = 0.00006/0.00005, *p* = 0.00016/0.00033, *p* = 0.00047/0.0069, *p* = 0.00005/0.00011, respectively). Moreover, ANOVA results showed that the differences in ZON concentration in infected tissue of cultivars Hanseat/Arina and the control plants at 168 h and two weeks were highly statistically significant (e.g., *p* = 0.00027/0.00028 and *p* = 0.00006/0.00001, respectively).

### 2.2. Generation of Superoxide Anion

**Figure 2:**
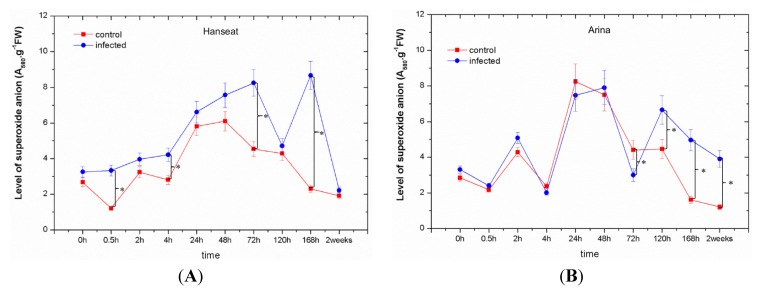
The effect of pathogenic fungus *Fusarium graminearum* on the generation of superoxide anion radical in ears of a susceptible wheat cv. Hanseat (**A**) and resistant cv. Arina (**B**). Significant differences (*p* < 0.005) were observed between control and infected ears.

Note: * significant differences on figures using asterisks are shown.

In the period from 0 to 72 h after inoculation in ears of wheat cv. Hanseat, a marked post-infection increase was observed in the generation of superoxide anion (O_2_^•−^), while starting from 120 h after inoculation it fluctuated (Figure 2A). Moreover, in the susceptible cv. Hanseat at all time points after inoculation a higher post-infection level of O_2_^•−^ generation was found in comparison to the control. In turn, in the wheat resistant cv. Arina up to 2 h after inoculation a higher level of O_2_^•−^ was recorded than in the control and next at later time points after inoculation with *F. graminearum*, *i.e.*, at 120, 168 h, and in week two after inoculation, O_2_^•−^ generation level was markedly higher than in the control (Figure 2B). It is of interest that at 24 and 48 h after inoculation a strong increase was found in the generation of O_2_^•−^, both in the control and in inoculated ears of wheat cv. Arina, whereas at 72 h a marked reduction of O_2_^•−^ was recorded in these tissues, while in inoculated ears the concentration of O_2_^•−^ was lower than in the control. Starting from 120 h after inoculation, the post-infection level of O_2_^•−^ was much higher than in the control. The significant differences in the level of superoxide anion were observed among the experimental variants as analyzed by ANOVA. ANOVA results showed that the differences in the concentration of O_2_^•−^ in infected tissue of cultivar Hanseat and the control plants at 0.5, 4, 72, and 168 h were highly statistically significant (e.g., *p* = 0.0001, *p* = 0.0011, *p* = 0.0007 and *p* = 0.0005), while in infected tissue of cultivar Arina and the control plants at 72, 120, 168 h, and two weeks they were highly statistically significant (e.g., *p* = 0.005, *p* = 0.00016, *p* = 0.00006 and *p* = 0.00009, respectively).

### 2.3. Generation of Semiquinone Radicals

**Figure 3:**
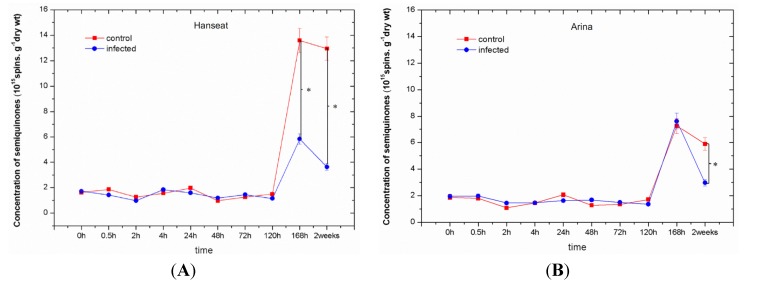
The effect of pathogenic fungus *Fusarium graminearum* on the generation of semiquinone radicals in ears of a susceptible wheat cv. Hanseat (**A**) and resistant cv. Arina (**B**). Significant differences (*p* < 0.005) were observed between control and infected ears.

Note: * significant differences on figures using asterisks are shown.

Levels of semiquinone radicals detected by electron paramagnetic resonance (EPR) increased at later time points of culture both in cv. Hanseat and Arina; however, in inoculated ears of wheat it was lower than in the control (Figure 3), except for 168 h after inoculation in ears of cv. Arina. Moreover, in the period from 0 to 120 h of culture in both wheat cultivars, Hanseat and Arina, slight fluctuations were observed in the concentration of semiquinone radicals both in the control and in inoculated tissues. However, the range of generation of these radicals in 168-h and two-week-old control tissues in the susceptible cv. Hanseat was two-fold greater than in the resistant cv. Arina. ANOVA results showed that the differences in concentrations of semiquinone radicals both in the control and in inoculated tissues were highly statistically significant. ANOVA results showed that the differences in semiquinone radical concentrations in infected tissue of the susceptible cv. Hanseat and the control plants at 168 h and two weeks were highly statistically significant (e.g., *p* = 0.0027 and *p* = 0.00064, respectively), while in the infected tissue of the resistant cv. Arina and the control plants at two weeks they were highly statistically significant (e.g., *p* = 0.00378).

### 2.4. Analysis of Disease Symptoms and the Level of Ergosterol

**Figure 4:**
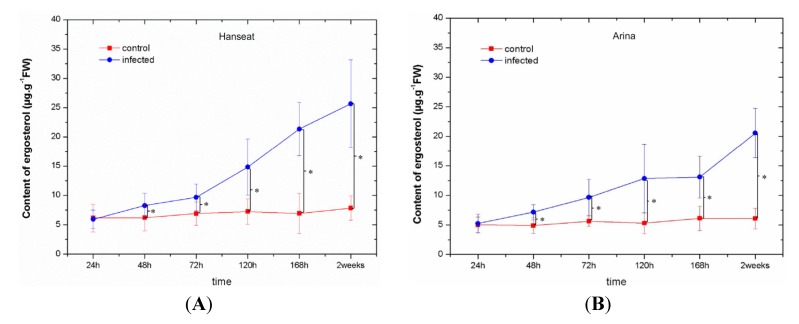
The level of ergosterol as a fungal growth indicator in ears of a susceptible wheat cv. Hanseat (**A**) and resistant cv. Arina (**B**). Significant differences (*p* < 0.05) were observed between control and infected ears.

Note: * significant differences on figures using asterisks are shown.

Table 1 showed disease development in ears of the susceptible wheat cv. Hanseat and resistant cv. Arina after inoculation with *Fusarium graminearum*. From 120 h after inoculation a pronounced severity of disease development was observed in ears of the susceptible wheat cv. Hanseat, it was stronger than in resistant cv. Arina. Symptoms first began as water-soaked brownish spots at the base of the glumes and ultimately turned into bigger brown discolorations. Moreover, masses of black spores occurred along the base of the glumes or over the infected head. In addition, from 120 h after inoculation the level of ergosterol in infected tissue of the susceptible wheat cv. Hanseat was higher than the resistant cv. Arina (Figure 4). ANOVA results showed that the differences in ergosterol concentration in infected tissue of cultivar Arina/Hanseat and the control plants at 48, 72, 120, 168 h, and two weeks were highly statistically significant (e.g., *p* = 0.03293/0.14943, *p* = 0.02159/0.06088, *p* = 0.01926/0.00921, *p* = 0.00777/0.00048 and *p* = 0.0001/0.00091, respectively).

**Table 1:** Disease development in ears of a susceptible wheat cv. Hanseat and resistant cv. Arina after inoculation with *Fusarium graminearum* (− lack of disease symptoms, + severity of disease symptoms, *i.e.*, strong discolorations and browning of ears where +++++ bigger brown discolorations in over 50% ears, sometimes black spores were found along ears and + single, light brown discolorations).

Time after inoculation	Disease development			
	Arina - Resistant		Hanseat - Susceptible	
	Control	Infected	Control	Infected
24 h	−	−	−	−
48 h	−	−	−	+
72 h	−	++	−	++
120 h	−	++	−	++++
168 h	−	+++	−	++++
2 weeks	−	++++	−	+++++

## 3. Discussion

This study investigated the interdependence between the level of oxidative stress and contents of mycotoxins, such as deoxynivalenol and zearalenone, in ears of two wheat cultivars, *i.e.*, susceptible Hanseat and Arina, resistant to a pathogenic fungus *Fusarium graminearum*. Our objective was to understand the difference in the invasion process of the host plant by the pathogen and the differential defense response in the resistant and susceptible cultivars. We detected changes in the redox status of cells in wheat ears, associated with the generation of superoxide anion and semiquinone radicals accompanying the accumulation of mycotoxins produced by the pathogenic fungus *F. graminearum*. The pathogenic fungus *F. graminearum* produces a range of sesquiterpenoid mycotoxins, including several types of B trichothecenes, such as DON and its acetylated derivatives 15Ac-DON and 3Ac-DON, which are required for full virulence on wheat ears [28,29,30,31]. Bin-Umer and co-workers reported that trichothecene toxins can inhibit mitochondrial translation independent of their effects on cytosolic translation and mitochondrial membrane integrity [32].

As a consequence of contact between the pathogen and the plant cell, biochemical reactions are initiated, limiting development of infection and disease. The first step of defense is the rapid generation of free radicals, including reactive oxygen species (ROS) and the activation of pre-existing components, such as the liberation of toxic compounds (e.g., phenolics and subsequent oxidative reactions) [33]. Thus, in the present study, in wheat ears of the susceptible cultivar we observed a marked post-infection increase in the generation of the superoxide anion radical (O_2_^•−^) in the period from 0 to 72 h after inoculation (Figure 2A). Additionally, its level at all time points after inoculation was higher than in the control. In turn, in wheat cv. Arina a markedly greater level of O_2_^•−^ generation in relation to the control was recorded at 2 h after inoculation and next at later time points after inoculation, *i.e.*, at the phase of disease development (Figure 2B). The difference in the level of O_2_^•−^ generation between the resistant wheat cultivar and the susceptible cultivar was connected with the earlier reduction in the generation of O_2_^•−^ in the resistant cultivar. In the resistant cultivar Arina fluctuations were observed in its generation *versus* time after inoculation. Perhaps this is related to the capture of electrons originating from superoxide anion (O_2_^•−^) by semiquinones.

Although superoxide anion (O_2_^•−^) is the proximal product generated, the more stable hydrogen peroxide (H_2_O_2_) species is detected in many studies [34]. Oxidative burst could have a direct effect on the pathogen or the defenses because of its reactivity. ROS could directly kill the pathogen, especially in the case of the more reactive species such as hydroxyl radicals [35]. ROS could also contribute to the establishment of physical barriers at the large papillae that are formed at the site of interaction of many pathogens by cross linking of cell wall glycoproteins [36] or via oxidative cross-linking of precursors during the localized biosynthesis of lignin and suberin polymers [37]. Gupta and co-workers reported that ROS are known to play pivotal roles in pathogen perception, recognition, and downstream defense signaling [38]. However, how these redox alarms coordinate, *in planta*, into a defensive network is still intangible.

Published literature sources comprise reports concerning modulation of the cellular redox status by mycotoxins produced by pathogenic fungi, but it is mainly in cells of animals and the human body, while there are scarce studies showing the above dependencies in plant cells. For example, recent studies showed modulation of the cellular redox status in human body cells by toxins of a pathogenic fungus, such as the *Alternaria* [39]. Therefore, the mycotoxins alternariol (AOH) and alternariol monomethyl ether (AME) were found to modulate the redox balance of HT29 cells from the human body, but without an apparent negative effect on DNA integrity. Additionally, Arunachalam and Doohan reported that trichothecene mycotoxins inhibit eukaryotic protein synthesis and are toxic to plants, humans and farm animals [40]. At the cellular level, they induce oxidative stress cell-cycle arrest and apoptosis, and affect membrane integrity. In animals, trichothecenes can be either immunostimulatory or immunosuppressive and induce apoptosis *via* mitochondria-mediated or -independent pathways. In turn, in plants trichothecenes induce programmed cell death via production of reactive oxygen species and they can induce genes involved in oxidative stress, cell death, and plant defense signaling. Studies by Gilchrist revealed a connection of mycotoxins between plants and animals in apoptosis and ceramide signaling [41]. Dobosz and co-workers showed also a relationship between the increase of the free form of salicylic acid (SA), free radical (FR) concentration, and propagation of *F. proliferatum* and *F. oxysporum* as a consequence mycotoxin formation, such as moniliformin and fumonisin B_1_ in infected plants of *Asparagus officinalis* [42]. In plants, the use of the *Arabidopsis* model system to understand molecular events in trichothecene-induced phytotoxicity has identified the involvement of MAPK signaling pathways and downstream transcription factors that manifest the toxicity effects [43,44]. Additionally, Desmond and co-workers demonstrated that infusion of wheat leaves with DON induced hydrogen peroxide production within 6 h, followed by cell death within 24 h that was accompanied by DNA laddering, a hallmark of programmed cell death [45].

In this study with an enhanced post-infection generation of O_2_^•−^ (Figure 2) a marked increase was observed in the contents of mycotoxins, such as DON and ZON; however, the highest accumulation of these toxins was recorded at the late period after inoculation in ears of both wheat cultivars, *i.e.*, at the disease development phase (Figure 1, Table 1). Up to 120 h after inoculation in ears of both wheat cultivars, the level of DON was higher than that of ZON, while at later time points (168 h and two weeks after inoculation) the resistant cultivar was characterized by a lower accumulation of DON than the sensitive cultivar. In parallel, in the resistant cultivar the development of disease symptoms (necrotic changes, discoloration of tissue) was limited (Table 1) and the level of ergosterol was lower than in the sensitive cultivar (Figure 4). Ergosterol (ERG) is a specific component of the fungal cell membrane [46]. It is also present in membranes in the cell walls and mitochondria in some yeasts, but is not produced in significant quantities by higher plants, rust fungi, or phycomycetes, hence, it can be used as a tool to estimate fungal biomass from any kind of mixtures [47,48]. A good positive correlation has been established between ergosterol content and fungal growth in other studies [49,50,51,52,53].

At the same time in this study, next to the increased generation of O_2_^•−^, which may be one of the lines of defense against *F. graminearum*, the concentration of free radicals, such as semiquinones was also determined (Figure 3). These free radicals detected in ears of wheat give signals characterized by *g*-value of 2.0037−2.0039 ± 0.0005, similarly as in previous reports [6,54,55,56,57], indicating that they are semiquinone-derived radicals.

It should also be mentioned that quinones, which represent the largest group of redox cycling compounds, are particularly active in ROS generation. Semiquinone radicals exhibit high reactivity and cytotoxicity and are formed during the oxidation of phenols by phenolases, peroxidases, and also by polyphenol oxidase activity. Moreover, the semiquinone radicals analyzed in this study using EPR spectrometry are among the relatively stable radicals. These oxidized phenolic species have an enhanced antimicrobial activity and thus may be directly involved in stopping pathogen development. During the pathogen-plant interaction, oxidation processes are stimulated, which enhances the effectiveness of defense mechanisms [6,10,11,56].

Measurements of semiquinone radicals using electron paramagnetic resonance (EPR) showed that the level of these radicals, in the period from 0 to 120 h, both in the control and in the infested ears of the sensitive and resistant cultivars showed fluctuations and ranged from 0.9 to 2 × 10^15^ spins g^−1^ dry weight. In turn, at the time points the concentration of these radicals increased rapidly in tissues both in cv. Hanseat and Arina, although, in infested ears of wheat, it was generally lower than in the control. We assume that the lower level of these radicals in relation to the control may indicate their involvement in the stimulation of defense mechanisms connected with strengthening of cell walls. It is also possible that these radicals in plant cells may be incorporated into polymers, such as lignins and by combining with reactive free radicals that propagate depolymerization through the lignin matrix, these protective free radicals could prevent the breakdown of associated cell walls [11,58]. Additionally, in the resistant cultivar the concentration of semiquinone radicals was lower than in the susceptible cultivar (Figure 3).

Summing up, recorded results indicate that the accumulation of mycotoxins produced by *F. graminearum* in ears of winter wheat was accompanied by a markedly enhanced generation of superoxide anion as an indicator of oxidative stress. A lower level of semiquinone radicals at later time points after inoculation may probably indicate their incorporation into polymers, e.g., such as lignins, by bonding with reactive oxygen species especially superoxide anion (O_2_^•−^) and, thus, strengthen the cell wall. The resistant cultivar was characterized by a lower level of semiquinone radicals than the sensitive cultivar especially at the late phase after inoculation. Development of disease was inhibited in the resistant cultivar and ergosterol content was lower than in the sensitive cultivar. Additionally, in the resistant cultivar production of the mycotoxin DON and the level of generation of superoxide anion (O_2_^•−^) at the late phase after inoculation was lower than in the sensitive cultivar.

## 4. Experimental Section

### 4.1. Plant Material and Growth Conditions

Plant material comprised two popular winter wheat cultivars with different susceptibility to *Fusarium*, *i.e.*, —a susceptible cv. Hanseat and a resistant cv. Arina. The experiment was performed in Cerekwica (Central West Poland, 30 km northwest of Poznań), in the randomized complete block design in triplicate, with plot size of 1 m × 1 m. Seeds of both winter wheat cultivars were sown in three independent plots both in the control and infected *F. graminarum*. Both cultivars, *i.e.*, Hanseat (susceptible) and the resistant Arina, originate from the Plant Breeding Company in Poznań, Poland.

### 4.2. Fusarium Strain and Inoculum Preparation

*Fusarium graminearum* strain KF 2870 (elsewhere referred to as *F. graminearum*) was obtained from the Collection of Plant Pathogenic Fungi held by the Institute of Plant Genetics Polish Academy of Sciences, Poznan. The pathogen was incubated in the dark at 25 °C in Petri dishes (+9 cm diameter) on potato dextrose agar (PDA) medium (Difco; pH 5.5). After three weeks of growth the *F. graminearum* spore suspension was prepared. The spore suspension was obtained by washing the mycelium with sterile water and shaking with glass pearls. At mid-anthesis (Zadoks scale 65), 30 winter wheat heads of each replication were inoculated individually (by brushing) with the conidial suspension (2 × 10^6^ spores) isolate of *Fusarium graminearum* (KF 2870). Non-inoculated plots of the same genotypes were used as the control. Inoculated and control samples (heads) for the determination of superoxide anion and semiquinone radicals were collected at 0, 0.5, 2, 4, 24, 48, 72, 120, 168 h, and two weeks after inoculation. In turn, for the determination of mycotoxins and ergosterol content, and analyses of disease development plant samples were collected at 24, 48, 72, 120, 168 h and two weeks after inoculation. To evaluate the disease, 50 ears of control plants and plants infected with *F. graminearum* were collected for both varieties, *i.e.*, resistant and sensitive.

### 4.3. Standards, Chemicals, and Reagents

Deoxynivalenol, zearalenone, and ergosterol standards and organic solvents (HPLC grade) were purchased with a standard grade certificate from Sigma-Aldrich (Steinheim, Germany). All chemicals used for extraction and purification of mycotoxins were purchased from POCh (Gliwice, Poland). Water for the HPLC mobile phase was purified using a Milli-Q system (Millipore, Bedford, MA, USA).

### 4.4. Extraction and Purification Procedure for Mycotoxins

Samples of 10 g homogenized winter wheat ears were prepared for analyses. Both mycotoxins (ZON and DON) were extracted and purified according to the detailed procedure described by Wiśniewska *et al*. [19]. The eluate was evaporated to dryness at 40 °C under a stream of nitrogen. Dry residue was stored at −20 °C until HPLC analyses.

### 4.5. HPLC Analysis of Mycotoxins

The chromatographic system consisted of a Waters 2695 high-performance liquid chromatograph (Waters, Milford, PA, USA) with detectors:

- Waters 2996 Photodiode Array Detector with a Nova Pak C-18 column (300 mm × 3.9 mm) for DON (λ_max_ = 224 nm) analysis,
- Waters 2475 Multi λ Fluorescence Detector (λ_ex_ = 274 nm, λ_em_ = 440 nm) and a Waters 2996 Photodiode Array Detector with a Nova Pak C-18 column (150 mm ×3.9 mm) for ZON analysis.

Quantification of mycotoxins was performed by measuring the peak areas at retention time according to the relevant calibration curve. The presence of mycotoxins was confirmed by a comparison of retention times with the external standard and by co-injection of every tenth sample with mycotoxin standards. Limits of detection were 0.001 µg g^−1^ for ZON and 0.01 µg g^−1^ for DON.

### 4.6. Ergosterol Extraction, Purification, and HPLC Analysis

Plant samples (100 mg) were suspended in 2 mL methanol in a culture tube, treated with 0.5 mL of 2 M aqueous sodium hydroxide, and sealed tightly. Samples were irradiated twice in a microwave oven (370 W) for 20 s. After 15 min contents of cultures tubes were neutralized with 1 M aqueous hydrochloric acid, then 2 mL methanol were added and samples were extracted with n-pentane (3 × 4 mL). The combined pentane extracts were evaporated to dryness in a stream of nitrogen, before analysis dissolved in 1 mL of methanol and 20 µL of thus prepared mixture were analyzed by HPLC. The ergosterol separation was performed on a 3.9 mm Nova Pak C-18, 4 mm column with methanol:acetonitrile (90:10, v/v) as the mobile phase at a flow rate of 1.0 mL min^−1^. EGR was detected with a Waters 2996 Photodiode Array Detector (Waters Division of Millipore, Milford, MA, USA) set at 282 nm. The presence of ergosterol was confirmed by a comparison of retention times with the external standard and by co-injection of every tenth sample with an ERG standard. The detection limit was 0.01 µg g^−1^ and standard deviation was below 7%.

### 4.7. Determination of Superoxide Anion Radical Content

Determination of superoxide anion radical (O_2_^•−^) content in biological samples was based on its ability to reduce nitro blue tetrazolium (NBT) [59]. The superoxide anion was detected according to Mai and co-workers [57], ears of wheat (0.30 g fr. wt) were cut into fragments (3 mm × 3 mm) and immersed in 10 mM potassium phosphate buffer (pH 7.8) containing 0.05% NBT and 10 mM NaN_3_ in a final volume of 3 mL and incubated for 1 h at room temperature. After incubation, 2 mL of the reaction solution were heated at 85 °C for 15 min and rapidly cooled. The levels of O_2_^•−^ in ears of wheat were expressed as absorbance at 580 nm per 1 g of fresh materials (A_580_.g^−1^ fr. wt). The measurement was carried out in the Perkin Elmer Lambda 15 UV-Vis spectrophotometer (Norwalk, CT, USA).

### 4.8. Determination of Semiquinone Radicals

Samples of several g fresh weight of wheat ears were frozen in liquid nitrogen and lyophilized in a Jouan LP3 freeze dryer. The lyophilized material was transferred to EPR-type quartz tubes of 6 mm in diameter. Electron paramagnetic resonance measurements were performed with a Bruker ELEXSYS spectrometer operating at the X-band. The EPR spectra were recorded at room temperature as derivatives of microwave absorption. A magnetic field modulation of about 2 Gs and a microwave power of 5 mW were typically used for all experiments to avoid line saturation and deformation. EPR spectra of free radicals were recorded in the magnetic field range of 3000–3650 Gs and with 4096 data points. In order to determine the number of paramagnetic centers (free radicals) in the samples, the spectra were double-integrated and compared with the intensity of the monocrystal standard chromium-doped corundum (Al_2_O_3_:Cr^3+^) with a known spin concentration [6,11,55,57,60,61]. Before and after the first integration of the spectra, small background corrections were made to obtain a reliable absorption signal before the second integration. Double integration of the free radicals was performed separately and this value was subtracted from the value obtained for the full 3000–3650 Gs scan range integration. As samples placed in quartz tubes were of equal volume, but of different weights, EPR intensity data were recalculated per 1 g of dry sample.

### 4.9. Statistical Analysis

All determinations were performed in three independent experiments. Analysis of variance (ANOVA) was applied to verify whether means from independent experiments within a given experimental variant were significant. Data shown in the figures are means of triplicates for each variant and standard errors of mean (SE). In individual figures significant differences are shown using asterisks.

## 5. Conclusions

A marked increase was found for the contents of mycotoxins, such as deoxynivalenol and zearalenone, in ears of both wheat cultivars in relation to the time after inoculation.

1. The susceptible cultivar was characterized by a much greater accumulation of deoxynivalenol than the resistant cultivar.
2. In the susceptible cultivar a marked post-infection increase in O_2_^•−^ level was found up to 120 h after inoculation.
3. The level of O_2_^•−^ generation in infested ears of both wheat cultivars was generally greater than in the control.
4. An earlier reduction in the level of O_2_^•−^ generation with the time after inoculation was observed in the resistant rather than in the susceptible cultivar.
5. The concentration of semiquinone radicals, detected by EPR, increased at later culture times; however, in infested ears of wheat it was generally lower than in the control.
6. The resistant cultivar was characterized by a lower level of semiquinone radicals than the sensitive cultivar especially at the late phase after inoculation. It may probably indicate their incorporation into polymers, such as lignins, and strengthening of the cell wall.
7. Development of disease was inhibited in the resistant cultivar and ergosterol content was lower than in the sensitive cultivar.
8. Production of the mycotoxin DON and the level of generation of superoxide anion (O_2_^•−^) in the resistant cultivar at the late phase after inoculation was lower than in the sensitive cultivar.
